# Controlled supramolecular structure of guanosine monophosphate in the interlayer space of layered double hydroxide

**DOI:** 10.3762/bjnano.7.184

**Published:** 2016-12-06

**Authors:** Gyeong-Hyeon Gwak, Istvan Kocsis, Yves-Marie Legrand, Mihail Barboiu, Jae-Min Oh

**Affiliations:** 1 Department of Chemistry and Medical Chemistry, College of Science and Technology, Yonsei University, Wonju, Gangwondo, 26493, Republic of Korea,; 2 Adaptive Supramolecular Nanosystems Group, Institut Européen des Membranes, University of Montpellier/ENSCM/CNRS 5635, Pl. Eugène Bataillon, CC 047, 34095 Montpellier, Cedex 5, France

**Keywords:** guanosine monophosphate, hydrogen bond, layered double hydroxide, ribbon structure, supramolecular assembly

## Abstract

Guanosine monophosphates (GMPs) were intercalated into the interlayer space of layered double hydroxides (LDHs) and the molecular arrangement of GMP was controlled in LDHs. The intercalation conditions such as GMP/LDH molar ratio and reaction temperature were systematically adjusted. When the GMP/LDH molar ratio was 1:2, which corresponds to the charge balance between positive LDH sheets and GMP anions, GMP molecules were well-intercalated to LDH. At high temperature (100 and 80 °C), a single GMP molecule existed separately in the LDH interlayer. On the other hand, at lower temperature (20, 40 and 60 °C), GMPs tended to form ribbon-type supramolecular assemblies. Differential scanning calorimetry showed that the ribbon-type GMP assembly had an intermolecular interaction energy of ≈101 kJ/mol, which corresponds to a double hydrogen bond between guanosine molecules. Once stabilized, the interlayer GMP orientations, single molecular and ribbon phase, were successfully converted to the other phase by adjusting the external environment by stoichiometry or temperature control.

## Introduction

Since Bernal [1] and Cairns-Smith [2] suggested that layered clays contributed to the origin of life by affecting the conformation of prebiotic molecules in ancient environment, many scientists have begun to focus their research on the molecular arrangement of biological substances in the interlayer space of layered inorganics [3–4]. In those investigations, monomeric prebiotic molecules such as amino acid, nucleoside or nucleotide were intercalated and it was suggested that the geometrical restriction of molecules under the confined interlayer space of inorganics would aid the polymerization of biological molecules.

Mann et al. intercalated two kinds of amino acids, aspartate and glutamate, into layered double hydroxide (LDH) to synthesize bioinorganic nanocomposites [5]. Post-synthetic thermal treatment resulted in polymerization of amino acids in LDH. It was also reported that amino acids such as arginine and glutamate were polymerized via peptidic condensation both on the surface and in the interlayer space of clay [6]. In that literature, peptidic condensation of amino acids was determined to favor heteropeptide rather than homopeptide. Besides polymerization of amino acids, it was reported that abiotic oligomerization of RNA nucleotides was catalyzed by montmorillonite clay [7]. In the presence of clay, the length of polymerized RNA oligonucleotides was three times longer than without clay.

Among biological molecules, guanosine derivatives are known to have various supramolecular assembly routes through intermolecular interactions. For instance, telomere in chromosome consists of stacks of guanosine quartets (G4), in which four guanosines are linked through hydrogen bonds in the presence of K^+^ ions. There have been many attempts to synthesize guanosine-based supramolecular assemblies such as ribbon or quadruplex. Spada et al. verified that two kinds of ribbon structures could be obtained by self-assembly of lipophilic deoxyguanosine derivatives through hydrogen bonding [8]. They suggested those supramolecular nucleoside structures could be utilized for molecular nanowire fabrication or molecular electronics. Davis et al. prepared stable G4 hydrogels utilizing guanosine, potassium and borate [9]. Those hydrogels exhibited specific binding ability towards cationic dyes or nucleoside through noncovalent interactions. Due to its characteristic structure having K^+^ in the center, G4 quartets can be considered as scaffolds for artificial ion channels. We have reported a stable G4 membrane film utilizing guanosine and bis(3-aminopropyl)polytetrahydrofuran [10]. These G4 membranes showed potential in Na^+^/K^+^ artificial ion channels.

Inspired by the above reports claiming that i) intermolecular interactions of biomolecules are strongly affected by the confined geometry of layered clays and that ii) guanosine derivatives form various supramolecular assemblies, depending on the chemical environment, we hypothesized that the orientation of guanosine derivatives can be controlled in the restricted nanospace of layered inorganics. In this study, we utilized layered double hydroxides (LDH), which are composed of positively charged layers (Mg_2_Al(OH)_6_^+^) with equivalent area per charge ≈25 Å^2^/(+) and charge compensating interlayer anions [11–12]. As it is known that interlayer anions in LDH can be easily exchanged by external anions, and that once introduced, anions are stabilized via electrostatic interaction [13–14], we utilized guanosine monophosphate (GMP) as a model molecule. First, we prepared LDHs with exchangeable anions and intercalated GMP through an ion exchange reaction. We evaluated the molecular conformation of GMP in the interlayer space of LDH by varying the GMP/Al^3+^ (in LDH) molar ratio and the reaction temperature. This was revealed that GMP molecules are separated or assembled into ribbons depending on the synthetic conditions. Furthermore, we confirmed that once stabilized GMP orientation in LDH could be converted to the other state by adjusting the physicochemical environment.

## Results and Discussion

**Figure 1:**
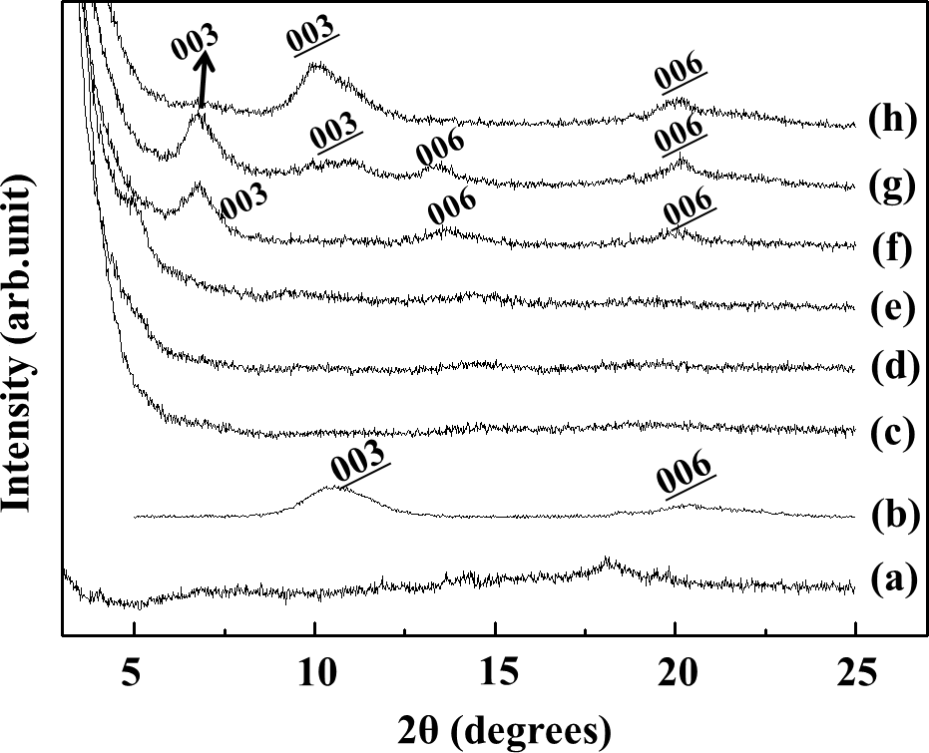
X-ray diffraction (XRD) patterns of products prepared by ion exchange between MgAl-LDH and GMP under controlled GMP/LDH molar ratios at 80 °C for 1 day. (a) GMP only, (b) MgAl-NO_3_-LDH, (c) 1:0.25, (d) 1:0.5, (e) 1:1, (f) 1:2, (g) 1:4, (h) 1:10 (GMP/LDH). Underlined Miller indexes are (00*l*) of NO_3_^−^ interlayer anion (MgAl-NO_3_-LDH). Miller indexes without underline are (00*l*) of GMP intercalated LDH.

In order to promote intercalation of GMP into the interlayer space of LDH, we set the reaction temperature at 80 °C, and varied the GMP/Al^3+^ (in LDH) ratio to 1:0.25, 1:0.5, 1:1, 1:2, 1:4 and 1:10. According to the adsorption isotherm, we found that GMP adsorption (or intercalation) to LDH follows Langmuir adsorption (Supporting Information File 1, Figure S1). The theoretical anionic exchange capacity (AEC) was satisfied at GMP/LDH ratio of 1.5:1, while the ideal stoichiometry GMP/LDH is 1:2 (note that GMP is a dianion and that the chemical formula of LDH is [Mg_2_Al(OH)_6_]^+^(NO_3_)^−^). Figure 1 shows X-ray diffraction (XRD) patterns of GMP/LDH (GL) hybrids prepared under various GMP/LDH molar ratios. At high GMP ratio (Figure 1c–e), the XRD patterns revealed amorphous structures, which was attributed to the disordered layer stacking of LDH due to the large quantity of organic moieties (GMP) as shown previously [15]. At GMP/LDH molar ratio of 1:2 and 1:4 (Figure 1f and Figure 1g, respectively), (00*l*) peaks shifted to lower angles as compared to pristine LDH, indicating ordered layer stacking with interlayer GMP moiety. However, excessive loading of LDH resulted in the decrease of intercalated peaks and increase of pristine peaks (Figure 1g,h). The optimum GMP/LDH ratio, yielding single-phased GMP intercalated LDH, was determined to be 1:2, which was expected from the stoichiometry according to charge neutralization. From the adsorption isotherm, the GMP/LDH ratio of 1:2 resulted in 90% of GMP uptake compared to theoretical AEC. The diameter of one GMP molecule is ≈1,000 Å^3^ and it is larger than the interlayer volume provided by the LDH (area per 2 charges (50 Å^2^) × gallery height (8–13 Å) ≈ 400–650 Å^3^). Thus, a GMP/LDH ratio of 1:2 would be sufficient to accommodate as many GMP molecules as possible in the LDH interlayer space. The d-spacing of GMP intercalated LDH was calculated to be 12.6 Å, which was the summation of the LDH layer thickness (≈4.8 Å) and the perpendicular arrangement of U-shaped GMP (≈7.8 Å).

**Figure 2:**
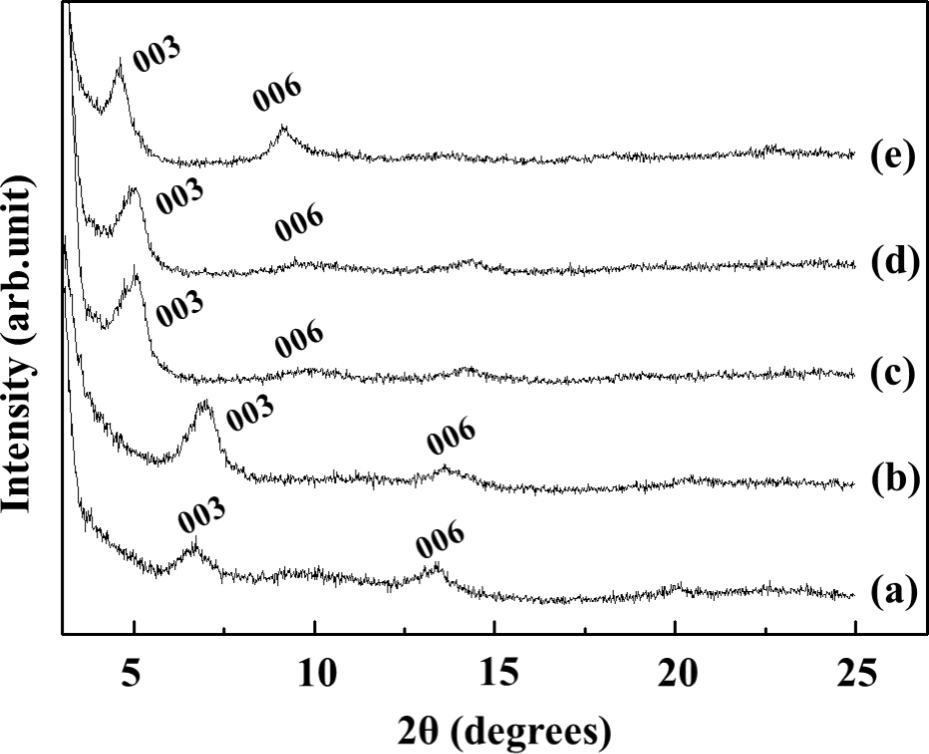
X-ray diffraction (XRD) patterns of GMP/LDH (GL) hybrid under controlled reaction temperature with GMP/LDH 1:2 for 1 day. (a) 100 °C, (b) 80 °C, (c) 60 °C, (d) 40 °C, (e) 20 °C.

In order to control the interlayer GMP arrangement, we fixed GMP/LDH to 1:2 and varied the reaction temperature. As shown in Figure 2, the GL hybrid obtained at 100 °C showed a similar XRD pattern (Figure 2b) compared with that obtained at 80 °C. On the other hand, those prepared at lower temperature (20, 40 and 60 °C) showed (00*l*) peaks shifted to lower angles (Figure 2c–e). The d-spacing values of the GL hybrid obtained at lower temperature was approximately 17.7 Å, which was 5.1 Å larger than for the single molecular orientation. Similar to the GMP adsorption at 80 °C, GMP uptake at 20 °C followed a Langmuir model. Although the Langmuir adsorption rate at 20 °C was slightly smaller than 80 °C, the overall adsorption patterns at the two temperatures were similar. Thus, the different interlayer space of GL hybrids at two different temperatures is thought to originate from interlayer molecular orientation of GMP. It is well known that guanosine moieties including GMP form various supramolecular structures result from its self-assembly. GMP makes quadruplex structures in the presence of specific metal as found in the telomere of chromosome [16]. Without metal cations, GMPs make ribbon-type structures through hydrogen bonding between H and electronegative sites (N or O). Two kinds of ribbon phases, I and II, have been reported [8].

**Figure 3:**
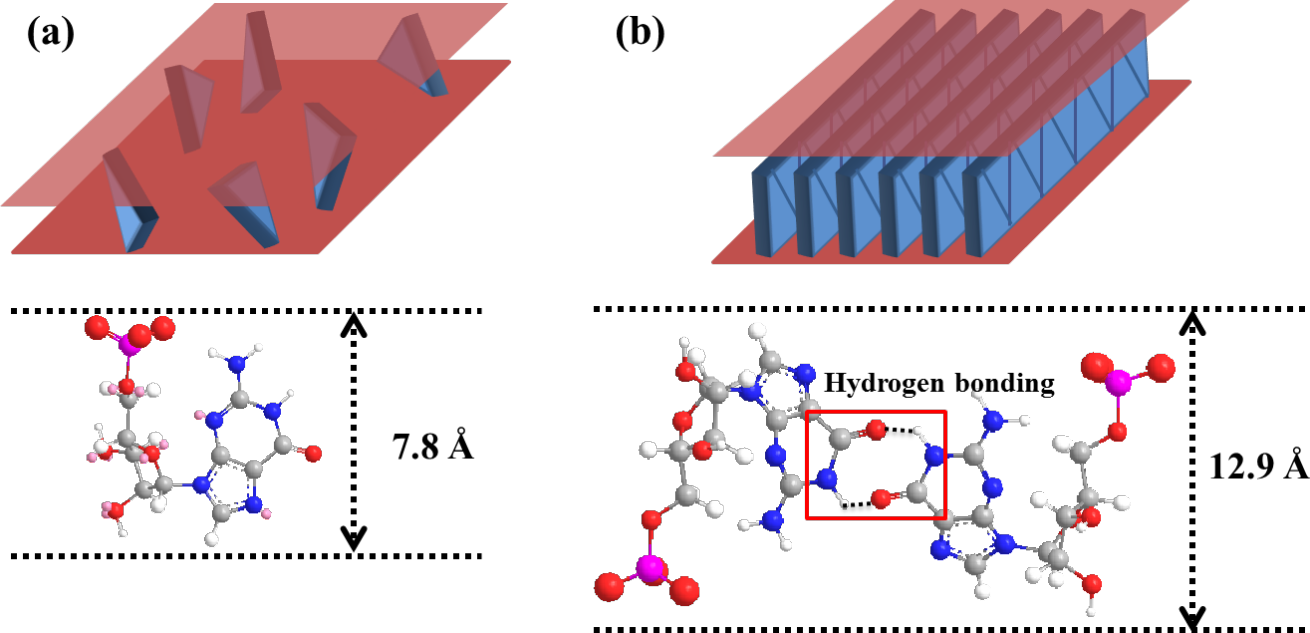
Schematic illustrations for interlayer structure of GMP/LDH hybrids according to molecular arrangement of GMPs: (a) single molecule arrangement (GL-S) and (b) ribbon II arrangement (GL-R).

Considering the molecular dimensions, the GMP intercalated to LDH layers at low temperature were thought to form in ribbon II supramolecular orientation (Figure 3b). At relatively high synthetic temperature, the formation of hydrogen bonds was disfavored and GMP existed as a single molecular arrangement (Figure 3a). Considering that double-helix DNA denatures to single-stranded DNA above 70 °C through hydrogen bonding breakage [17], the reaction temperature above 60 °C in this study might be sufficient to disrupt hydrogen bonding between GMPs. It was interesting to observe that the single molecular arrangement of GMP obtained at relatively high temperature was preserved between LDH layers after the material was cooled down to room temperature. Two kinds of GL hybrids with different GMP arranagements, single and ribbon, were named GL-S and GL-R, respectively.

**Figure 4:**
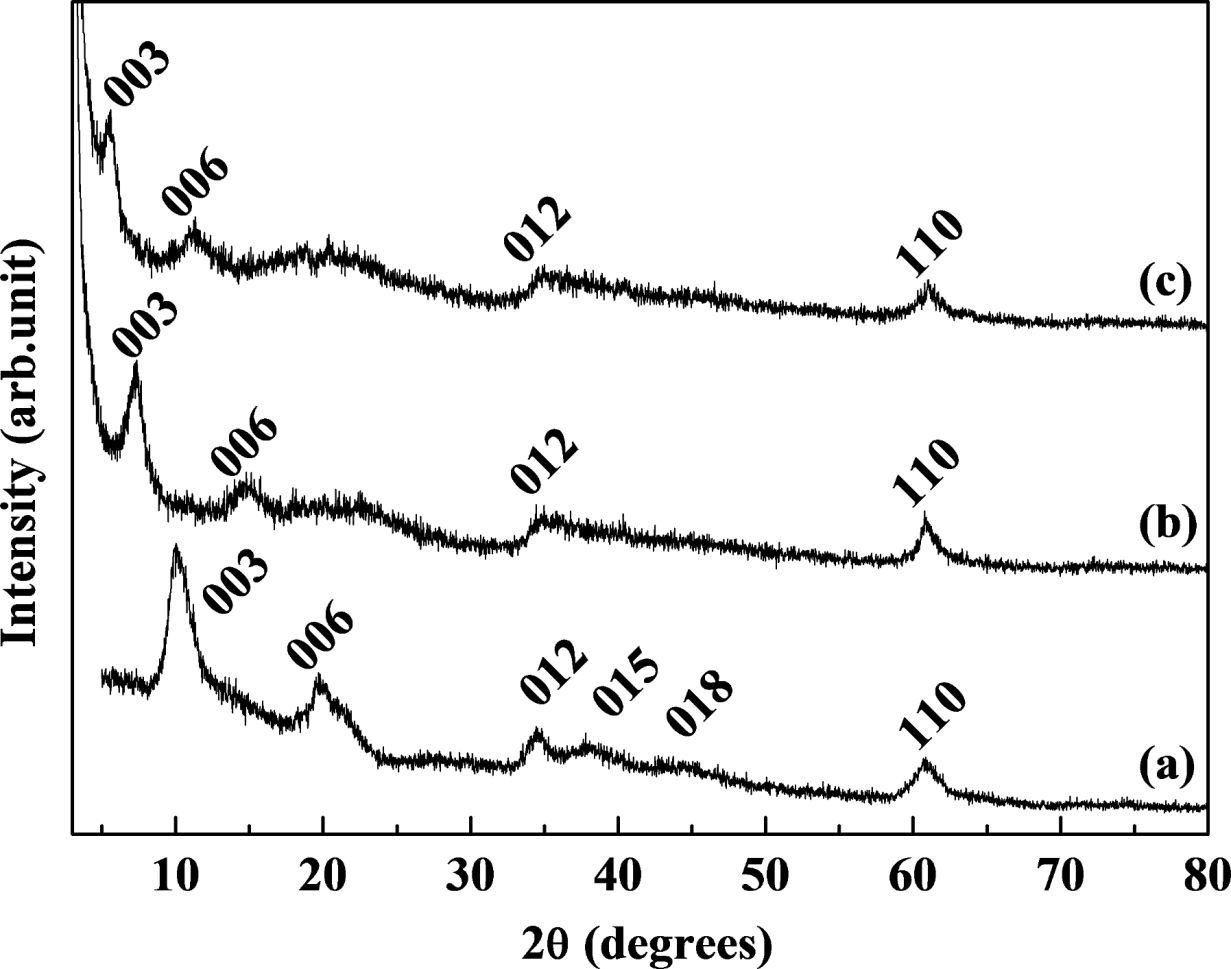
XRD patterns of (a) pristine MgAl-NO_3_-LDH, (b) GL-S and (c) GL-R.

Figure 4 shows powder XRD patterns of pristine LDH, GL-S and GL-R at the 2θ region 3–80°, showing clear lattice peaks at (012) and (110) for hybrids. This result showed that the lattice of LDH along *a*–*b*-plane direction was well preserved while there was lattice expansion along the *c*-axis resulting from molecular orientation of GMPs.

According to thermogravimetric analysis (TGA) and X-ray fluorescence (XRF) spectroscopy, the chemical formula of GL-S and GL-R were determined to be Mg_2.00_Al(OH)_6_(GMP)_0.37_(NO_3_)_0.26_·(H_2_O_interlayer_)(0.63H_2_O_surface_) and Mg_2.00_Al(OH)_6_(GMP)_0.42_(NO_3_)_0.16_·(H_2_O_interlayer_)(0.82H_2_O_surface_), respectively. Slightly larger GMP content in GL-R as compared to GL-S could explain the formation of the ribbon arrangement of GMP in the GL-R hybrid. The area of the LDH layer, Mg_2_Al(OH)_6_^+^, is approximately 25 Å^2^. Considering that the cross-sectional area of one GMP molecule is ≈60 Å^2^, GMP occupies 22 Å^2^ (60 Å^2^ × 0.37) and 25 Å^2^ (60 Å^2^ × 0.42), respectively for GL-S and GL-R. Thus, the GMP moiety in GL-S could exist separately without effective intermolecular interaction by GMP, while GMPs in GL-R packs to form a ribbon-type supramolecular arrangement.

**Figure 5:**
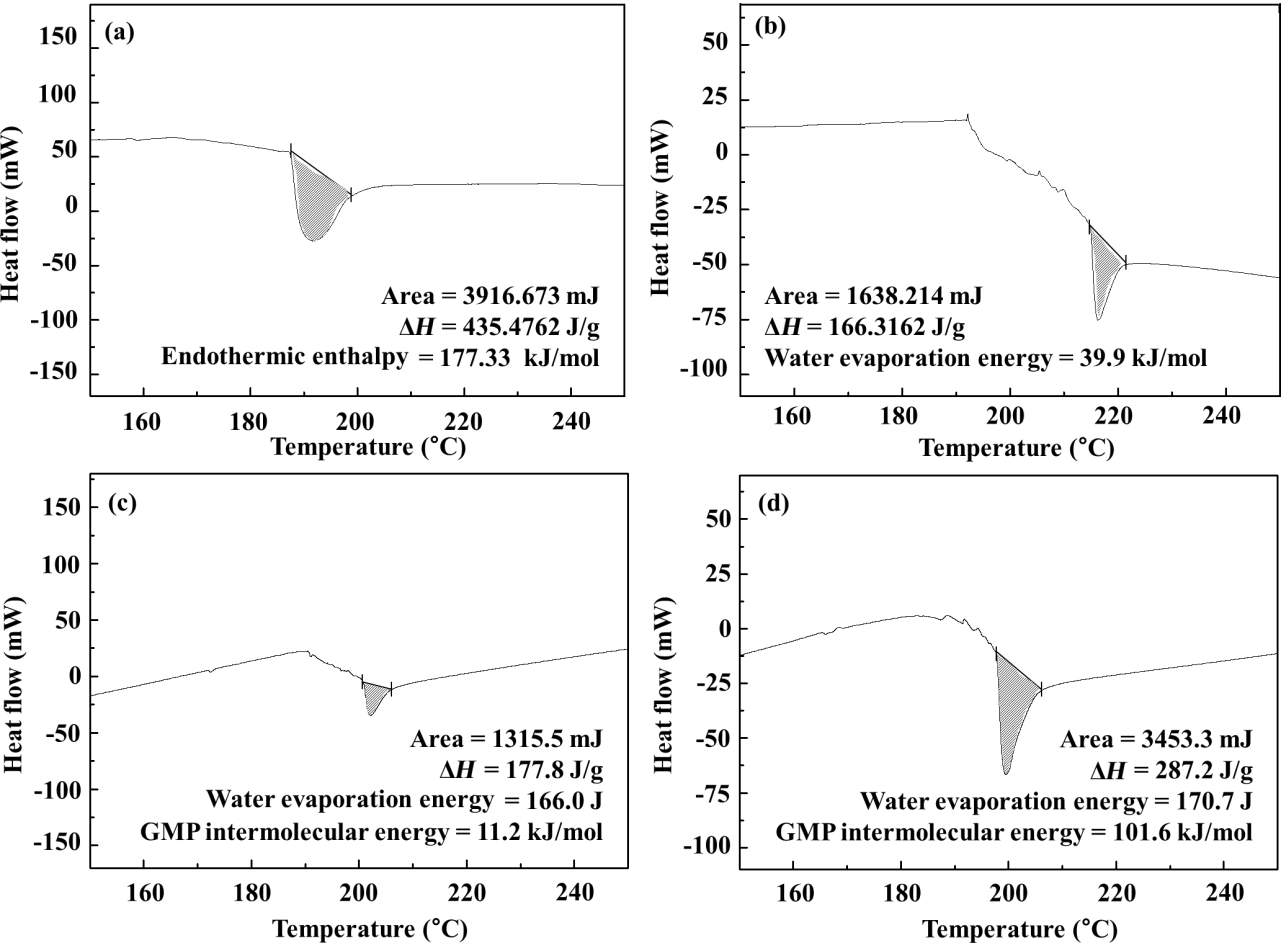
Differential scanning calorimetry (DSC) diagrams of (a) GMP powder, (b) pristine MgAl-NO_3_-LDH, (c) GL-S and (d) GL-R.

Differential scanning calorimetry (DSC) diagrams for both GL hybrids suggested the hydrogen bonding among GMP moiety in GL-R hybrid (Figure 5). For comparison, we carried out DSC measurements of GMP only, which showed 177.33 kJ/mol of endothermic enthalpy change at 191.28 °C, indicating strong hydrogen bonding among GMP molecules. Also, we verified that the DSC diagram of pristine LDH showed an endothermic peak at 216.23 °C with an enthalpy change of 39.9 kJ/mol, which exactly matches the calculated water evaporation energy (≈40 kJ/mol) of Mg_2_Al(OH)_6_(NO_3_)·H_2_O. Both hybrids showed a strong endothermic peak at around 200 °C, which was attributed to breaking intermolecular interaction of interlayer GMP as well as evaporating interlayer water molecules. Interlayer water was evaporated from the DSC of pristine LDH at around 210 °C with an enthalpy change of ≈40 kJ/mol, which was subtracted from the endothermic enthalpy of GL hybrids. The resulting intermolecular interaction between GMP was found to be 11.2 kJ/mol GMP and 101.6 kJ/mol GMP for GL-S and GL-R, respectively. The much higher energy for GL-R implied that there might be strong intermolecular interaction such as hydrogen bonding. According to the literature where hydrogen bonding energy between base pairs was simulated, double hydrogen bonded guanine–cytosine pair had 74–96 kJ/mol depending on bonding orientation [18]. Thus, the intermolecular energy of 101.6 kJ/mol (Figure 5d) might be attributed to the double hydrogen bonding between the GMP moiety as shown in Figure 3b. The DSC for GMP only (powder state) showed endothermic enthalpy of 177 kJ/mol GMP at 190 °C, implying that there was strong intermolecular interaction and possible formation of supramolecular structure in powder GMP.

**Figure 6:**
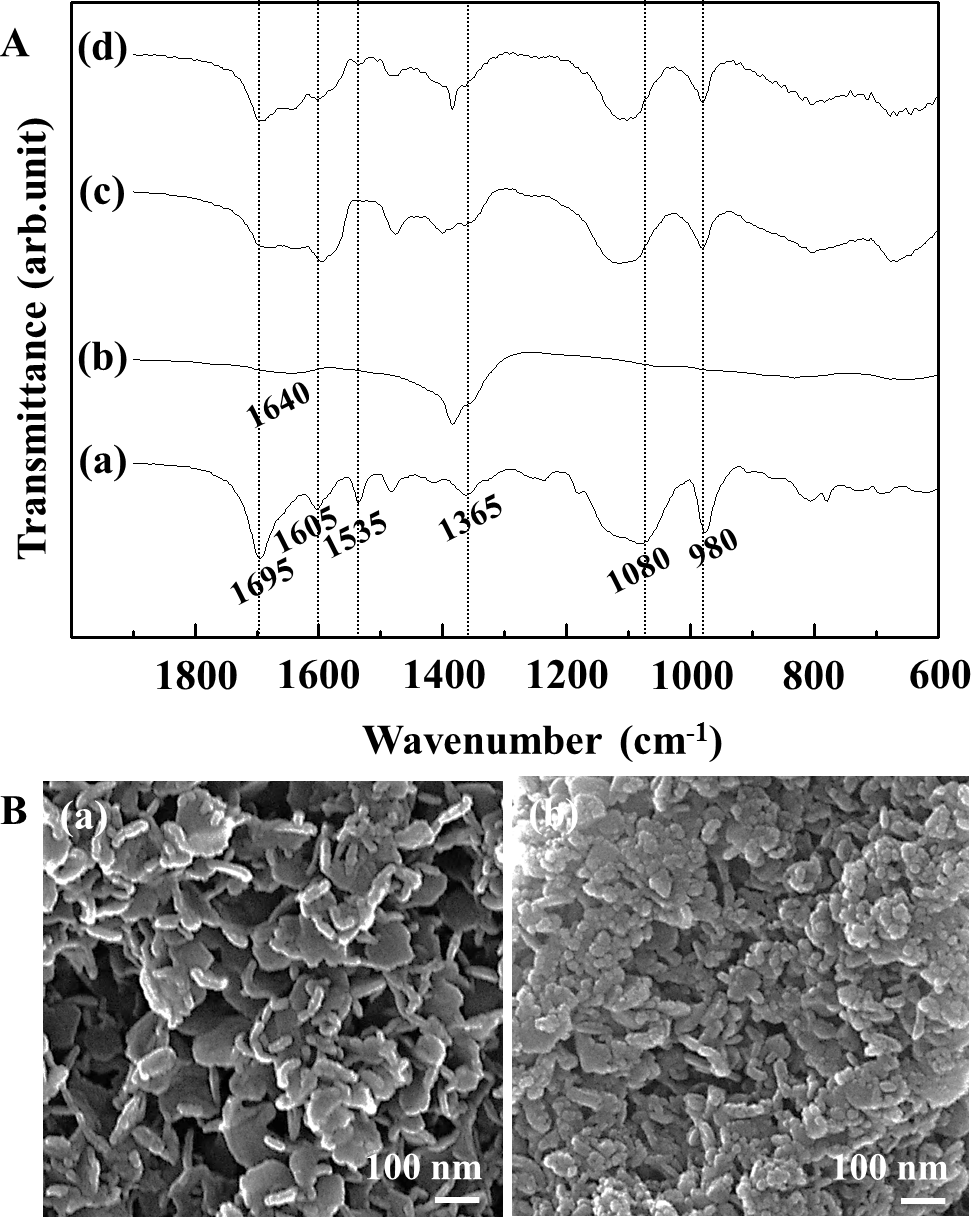
(a) Fourier transformed infrared (FTIR) spectra of GMP powder (line a), pristine MgAl-NO_3_-LDH (line b), GL-S (line c) and GL-R hybrid (line d). (b) Scanning electron microscopic (SEM) images of GL-S (image a) and GL-R hybrid (image b).

To identify the supramolecular assembly of GMPs in GL-R, we carried out ^13^C solid state nuclear magnetic resonance (NMR) utilizing a G4 quartet supramolecular assembly as the reference (Supporting Information File 1, Figure S2). The G4 quartet showed a single set of signals due to their symmetric structure. In GL-R, the signals for the quartet slightly shifted as well as the evolution of new signals. The result suggested that the supramolecular structure in GL-R was asymmetric and possibly ribbon-like. In order to verify that the intact structure of GMP is preserved after intercalation into LDH, we carried out Fourier transform infrared (FTIR) spectroscopy. As shown in Figure 6a, GMP (a) shows typical vibrational modes at 1695, 1605, 1535, and 1365 cm^−1^, which were attributed to C=O, C=N, pyrimidine/imidazole and imidazole vibration, respectively. Two bands at 1080 and 980 cm^−1^ resulted from PO_3_^2−^ antisymmetric stretching and PO_3_^2−^ symmetric stretching [19]. Both GL-S (Figure 6a, line c) and GL-R (Figure 6a, line d) showed similar IR bands compared with GMP only, although the bands in the range 1550–1800 cm^−1^ could not be separated in detail due to the merging with the δ-mode of interlayer water (1640 cm^−1^). Particle morphologies of GL hybrids are displayed in Figure 6b. Both images showed LDH particles with homogeneous size distribution. The average particle size was determined to be ≈90 nm and ≈50 nm for GL-S and GL-R, respectively. The slightly larger size of GL-S was attributed to the aging effect in inorganic particles [20], as GL-S was heated to 80 °C. Compared to the SEM image of the GMP reagent and pristine LDH (Supporting Information File 1, Figure S3), we verified that the particle morphology of both GL hybrids exhibited a typical pattern of organic moiety intercalated LDHs. Disk-shaped particles were agglomerated due to the particle-edge interaction between LDH particles [21].

**Figure 7:**
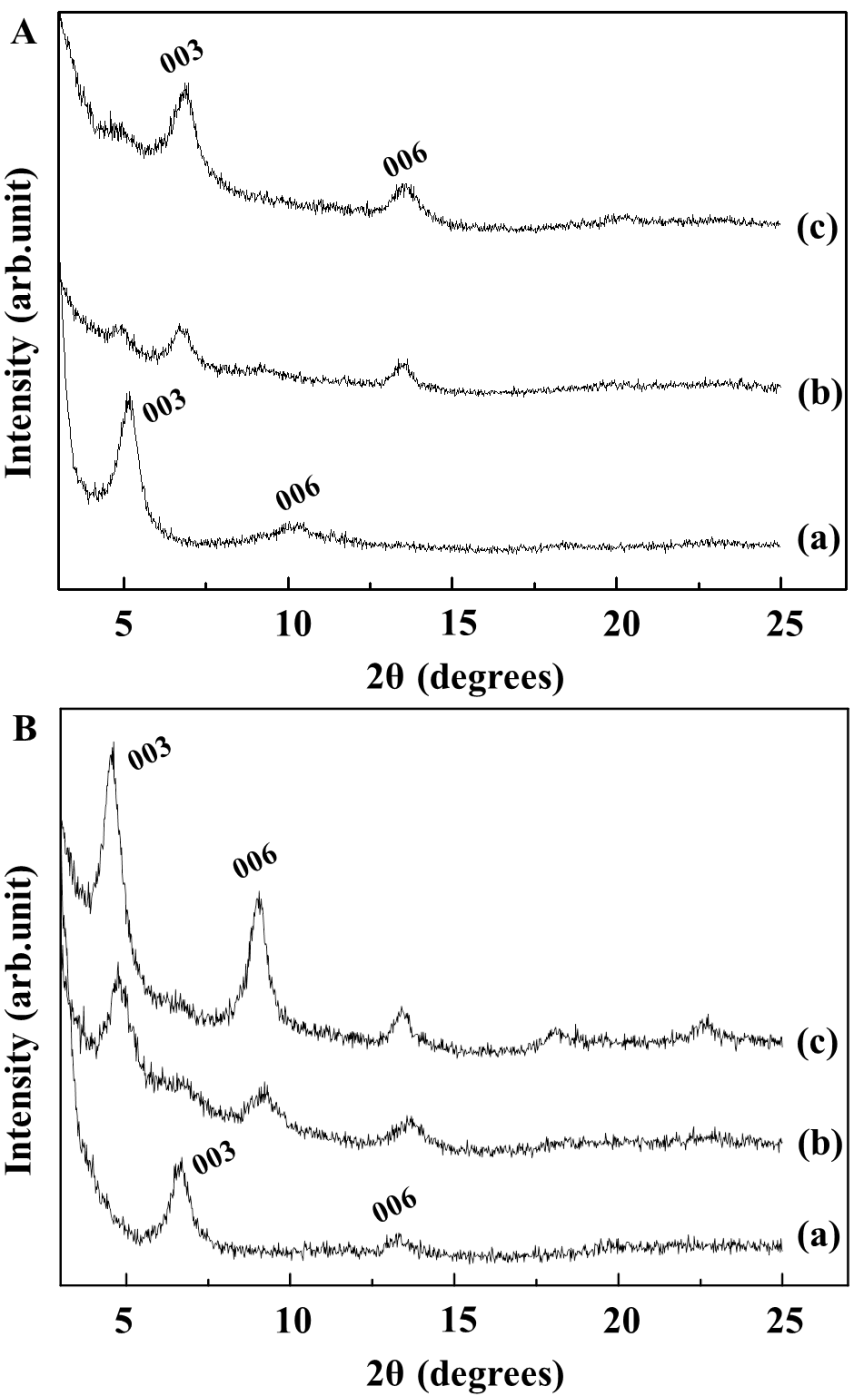
X-ray diffraction (XRD) patterns of GL hybrids obtained by interconverting interlayer GMP arrangement. (a): GL-R to GL-S and (b): GL-S to GL-R, for starting (line a), intermediate (line b) and final (line c) products.

We confirmed that interlayer arrangements of GMP molecules, such as single molecular or ribbon orientation, could be obtained according to specific synthetic conditions. As a further step, we tried to verify whether the interlayer arrangement could be converted to the other phase by adjusting the external conditions. The addition of LDH while heating was thought to convert GL-R to GL-S as the condition disrupts hydrogen bonding and provides more interlayer space, allowing molecules to be isolated. As shown in Figure 7a, one day of stirring at 80 °C with the addition of 1 equivalent LDH showed the evolution of the (003) peak at 7° (Figure 7a, line b), which corresponded to the interlayer space of single molecule arrangement. After two days, the (003) peak corresponding to the ribbon orientation almost disappeared and the peak at 7° became dominant, implying that the GL-R was converted into GL-S. In order to convert GL-S to GL-R, we applied a similar strategy. One equivalent of GMP was added in solution phase to the GL-S suspension, while the temperature was set to 20 °C. According to Figure 7b (line b), the intensity of (003) for GL-S decreases after one day of stirring and a new peak at 4.8°, which matched with that for GL-R, began to appear. After two days, we could obtain XRD patterns corresponding to GL-R. These results suggested that the surrounding conditions switch the stable interlayer molecular orientation of GMP.

## Conclusion

GMP was intercalated into the interlayer space of LDH (GL hybrids) and its molecular arrangement was controlled to form in either single molecular orientation or ribbon-type supramolecular assembly according to synthetic conditions. At high temperature (80 and 100 °C), GMPs were separated in single molecules and at low temperature (20, 40, and 60 °C), GMPs assembled in a ribbon structure. The intermolecular energy for GMP, ≈101 kJ/mol, suggested the possibility of double hydrogen bond between GMPs in GL-R. We also verified that, once stabilized, GL-S or GL-R could be converted to the other phases by adjusting synthetic conditions like stoichiometry and temperature. The stoichiometry between the LDH and GMP determined interlayer molecular packing patterns, which in turn influenced intermolecular interaction among GMP molecules. Furthermore, even in confined space, the temperature affected the formation and breakage of hydrogen bonds.

## Experimental

### Materials

Magnesium nitrate hexahydrate (Mg(NO_3_)_2_·6H_2_O) and aluminum nitrate nonahydrate (Al(NO_3_)_3_·9H_2_O) were purchased from Sigma-Aldrich (St. Louis, USA). Guanosine 5'-monophosphate disodium salt hydrate (C_10_H_12_N_5_Na_2_O_8_P·*x*H_2_O) was purchased from Tokyo Chemical Industry Co., Ltd. (Tokyo, Japan). Sodium hydroxide (NaOH) in pellet form was purchased from Daejung Chemicals & Metals Co., Ltd. (Siheung-si, Gyonggi-do, Korea).

#### Preparation of pristine MgAl-NO_3_-LDH

An aqueous solution in which Mg(NO_3_)_2_·6H_2_O (0.45 M, 45 mmol) and Al(NO_3_)_3_·9H_2_O (0.225 M, 22.5 mmol) were dissolved in decarbonated water was titrated with NaOH (0.75 M) solution up to pH ≈9.5. After a white-colored suspension was formed, the reaction was kept stirring for 24 h. The obtained MgAl-NO_3_-LDH sample was centrifuged and thoroughly washed with decarbonated water.

#### Preparation of GL hybrids

MgAl-NO_3_-LDH was dispersed in decarbonated water to achieve a ≈0.06 g/mL concentration. In order to intercalate GMP into LDH (GL hybrid), two parameters, the GMP/Al^3+^ (in LDH) molar ratio and reaction temperature, were controlled individually. For the molar ratio control experiment, 0.186 mmol of GMP (0.07582 g) was dissolved in 10 mL of decarbonated water at 80 °C. Then LDH suspensions of 0.25, 0.5, 1, 2, 4, and 10 equiv Al^3+^ were added to the GMP solution and stirred for one day at 80 °C under N_2_ atmosphere. For the temperature control measurement, a GMP/Al^3+^ (in LDH) molar ratio 1:2 was chosen and mixtures were reacted at 20, 40, 60, 80, and 100 °C, respectively, for one day. We found optimized preparation conditions for GL-S (GL hybrid with single molecular GMP orientation) and GL-R (GL hybrid with ribbon phase GMP structure) hybrid as follows. GMP/LDH molar ratio 1:2, reaction temperature 80 °C and two days reaction time for GL-S; GMP/LDH molar ratio 1:1, reaction temperature 20 °C and one day reaction for GL-R hybrid.

#### Conversion of interlayer GMP arrangement

In order to convert GL-R to GL-S, 1 equiv of LDH suspension was added to the GL-R suspension at 80 °C and was stirred for one day. For the reverse reaction (GL-S to GL-R), 1 equiv of GMP was added to the GL-S suspension at room temperature under stirring for two days.

#### Characterization

All the samples were thoroughly washed with decarbonated water and lyophilized before characterization. The adsorption isotherm experiment was carried out with a UV–vis spectrophotometer (UV–vis; Shimadzu UV-1800, Shimadzu Corporation, Kyoto, Japan) at λ_max_ = 253 nm. In order to identify the crystal structure of pristine LDH and to evaluate the lattice expansion of LDH along the *c-*axis upon GMP intercalation, X-ray diffraction patterns (XRD) were obtained with a Bruker D2 instrument with Ni-filtered Cu Kα radiation (λ = 1.5406 Å). The hydrogen bonding of the GL-R hybrid was verified by differential scanning calorimetry diagrams (DSC, PerkinElmer DSC 8000) with 5 °/min heating rate. To investigate the intact structure and intermolecular interaction of GMP in GL hybrids, Fourier transform-infrared spectra (FTIR, PerkinElmer, Spectrum One) with conventional KBr pellet method was carried out. The morphology of GL hybrids was observed by scanning electron microscopic (SEM) images obtained with an FEI QUANTA 250 FEG. For quantitative analysis, a thermogravimetric analyzer (TGA, SINCO STA S-1000) and X-ray fluorescence spectrometer (XRF, Thermo Scientific ARL QUANT’X) were used.

## Supporting Information

File 1 Adsorption isotherm, theoretical Langmuir plots, solid-state NMR spectra, and additional SEM images.
