# Supplementary material for: Controlled supramolecular structure of guanosine monophosphate in the interlayer space of layered double hydroxide
Source: Beilstein J Nanotechnol. 2016 Dec 6;7:1928–35. doi: 10.3762/bjnano.7.184 (PMC5238632; doi:10.3762/bjnano.7.184)
Supplement: File 1 — Adsorption isotherm, theoretical Langmuir plots, solid-state NMR spectra, and additional SEM images. [file Beilstein_J_Nanotechnol-07-1928-s001.pdf]

Supporting Information

for

Controlled supramolecular structure of guanosine monophosphate in the interlayer space of layered double hydroxide

Gyeong-Hyeon Gwak[1,§], Istvan Kocsis[2,§], Yves-Marie Legrand[2,§], Mihail Barboiu[2,*], and Jae-Min Oh[1,*]

Address: [1]Department of Chemistry and Medical Chemistry, College of Science and Technology, Yonsei University, Wonju, Gangwondo, 26493, Republic of Korea, and [2]Adaptive Supramolecular Nanosystems Group, Institut Européen des Membranes, University of Montpellier/ENSCM/CNRS 5635, Pl. Eugène Bataillon, CC 047, 34095 Montpellier, Cedex 5, France

Email: Mihail Barboiu* - mihail-dumitru.barboiu@univ-montp2.fr; Jae-Min Oh* - jaemin.oh@yonsei.ac.kr

* Corresponding author

[§]Equally contributing authors

Adsorption isotherm, theoretical Langmuir plots, solid-state NMR spectra, and additional SEM images

Adsorption isotherm of GMP on LDH

The isotherm showed typical Langmuir adsorption with high regression coefficient. The Qe value corresponding to the theoretical anionic exchange capacity (AEC) was found at GMP/LDH ratio ~1.5. The GMP molecules we used in this study are disodium salt form and also have two (-) charge sites per one molecule; GMP/LDH molar ratio (GMP/Al^{3+} (LDH)) of 0.5:1 would be the stoichiometry for GMP-LDH hybrid with maximum GMP intercalation. Thus three times more GMP molecules than intercalation stoichiometry is required for sufficient intercalation. The GMP/LDH molar ratio for hybridization in this manuscript is 1:1, at which 90% of GMP compared with AEC would be incorporated into LDH. This value was well corresponding to the partial intercalation of GMP into LDH for both GL-S and GL-R hybrids. (GL-S: $Mg_{2.00}Al(OH)_6(GMP)_{0.37}(NO_3)_{0.26}\cdot 1.63H_2O$, GL-R: $Mg_{2.00}Al(OH)_6(GMP)_{0.42}(NO_3)_{0.16}\cdot 1.82H_2O$). The partial intercalation was intentionally designed in our hybridization due to smaller interlayer volume provided by LDH (demanded area per two charge 50 $Å^2$ $\times$ interlayer distance 8~13 Å = 400~650 $Å^3$) than the dimension of one GMP molecule (approximately 1000 $Å^3$). In terms of energy transfer, the GMP intercalation (or adsorption) was considered endothermic

as the Langmuir adsorption rate (b) was larger at 80°C (b=4.08×10^{-4}) than at room temperature (b=3.24×10^{-4}).

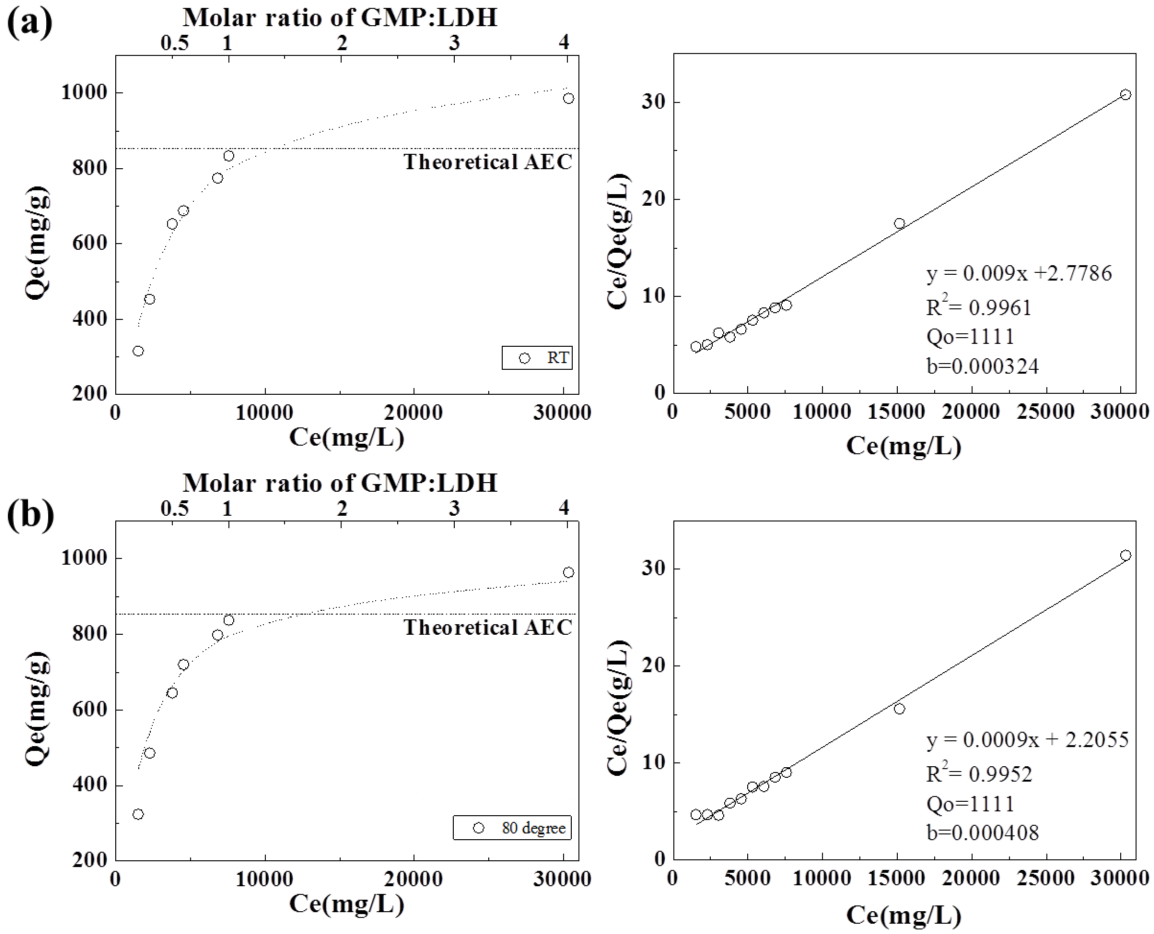

Figure S1: Adsorption isotherm (open circle) and theoretical Langmuir plot (dotted line) at (a) 20°C and (b) 80°C (left). Linear regression results for Langmuir fitting (right) with corresponding Langmuir coefficient..

Solid state NMR spectra

Nuclear magnetic resonance (NMR) spectrum of G4 showed single set of signals for the C2 (154 ppm) and C6 (161 ppm) due to their symmetric structure. In GL-R, the signals for the quartet structures slightly shifted as well as a new set of signals at

169-170 ppm that seem to present the split signals specific for the asymmetric ribbon organization. The NMR suggested that the hydrogen bonding in GL-R resulted in ribbon-like structure.

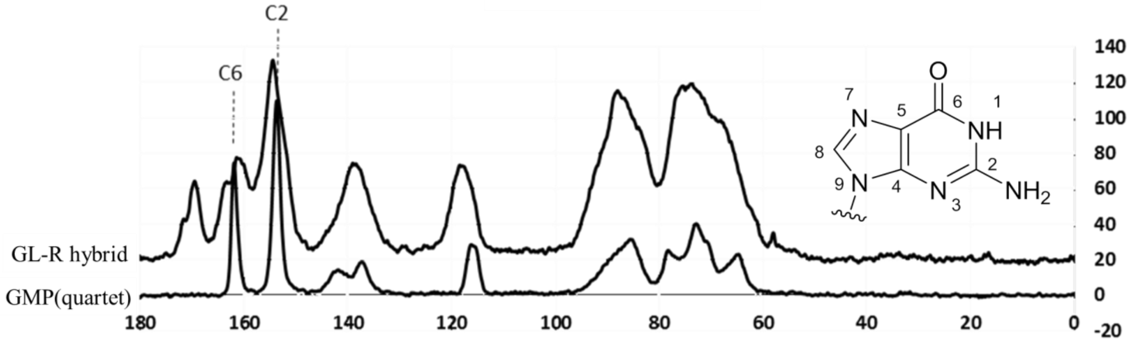

Figure S2: Solid state NMR spectra of GMP(quartet) and GL-R.

SEM images

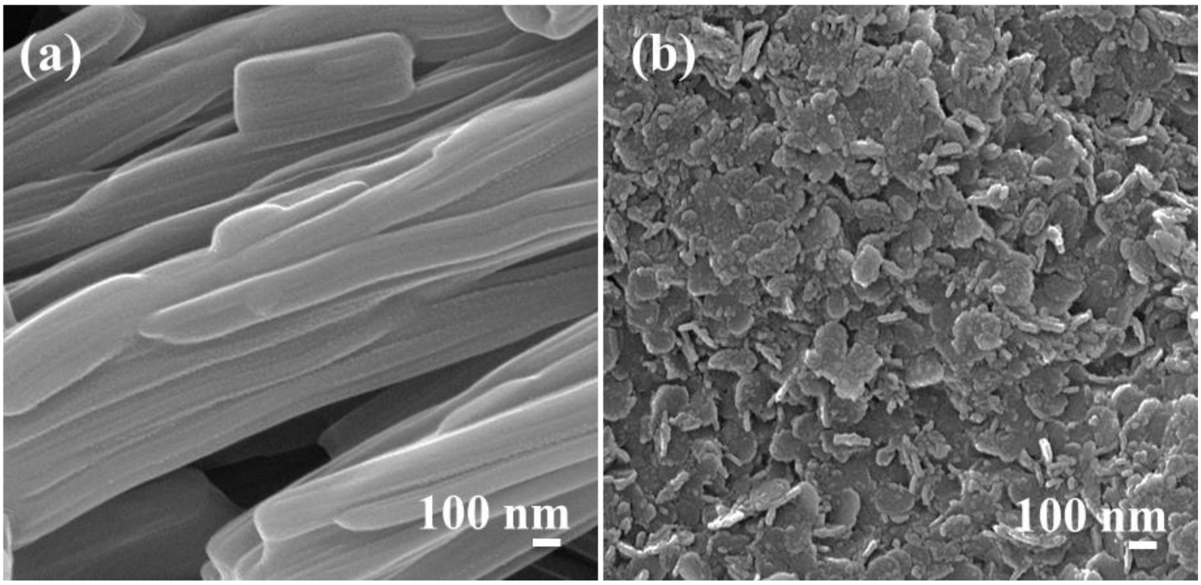

Figure S3: SEM images of (a) GMP reagent and (b) MgAl-NO_3-LDH pristine.
